# Why Is the Duration of Erythema Migrans at Diagnosis Longer in Patients with Lyme Neuroborreliosis Than in Those without Neurologic Involvement?

**DOI:** 10.3390/pathogens13020137

**Published:** 2024-02-01

**Authors:** Katarina Ogrinc, Petra Bogovič, Vera Maraspin, Stanka Lotrič-Furlan, Tereza Rojko, Andrej Kastrin, Klemen Strle, Gary P. Wormser, Franc Strle

**Affiliations:** 1 Department of Infectious Diseases, University Medical Centre Ljubljana, 1525 Ljubljana, Slovenia; katarina.ogrinc@kclj.si (K.O.); petra.bogovic@kclj.si (P.B.); vera.maraspin@kclj.si (V.M.); stanka.lotric@kclj.si (S.L.-F.); tereza.rojko@kclj.si (T.R.); 2 Institute for Biostatistics and Medical Informatics, University of Ljubljana, 1000 Ljubljana, Slovenia; andrej.kastrin@mf.uni-lj.si; 3 Department of Molecular Biology and Microbiology, Tufts University School of Medicine, Tufts University, Boston, MA 02111, USA; klemen.strle@tufts.edu; 4 Division of Infectious Diseases, New York Medical College, Valhalla, NY 10595, USA; gwormser@nymc.edu

**Keywords:** Lyme borreliosis, Lyme disease, erythema migrans, Bannwarth’s syndrome, borrelial meningoradiculoneuritis, Lyme neuroborreliosis, *Borrelia burgdorferi* sensu lato

## Abstract

In prior studies, the skin lesion erythema migrans (EM) was present for a longer time period before diagnosis of concomitant borrelial meningoradiculoneuritis (Bannwarth’s syndrome) compared to EM patients without neurologic symptoms. To determine if this observation pertains to other manifestations of Lyme neuroborreliosis (LNB), we compared EM characteristics in patients with borrelial meningoradiculoneuritis (n = 122) to those with aseptic meningitis without radicular pain (n = 72 patients), and to patients with EM but without neurologic involvement (n = 12,384). We also assessed factors that might impact duration. We found that the duration of EM at diagnosis in patients with borrelial meningoradiculoneuritis was not significantly different compared with those with LNB without radicular pain (34 vs. 26 days; *p* = 0.227). The duration of EM for each of these clinical presentations of LNB, however, was significantly longer than in patients with EM without LNB (10 days; *p* < 0.001). Contributing factors to this difference might have been that patients with LNB failed to recognize that they had EM or were unaware of the importance of not delaying antibiotic treatment for EM. In conclusion, the duration of the EM skin lesion in EM patients with LNB is longer than in patients with just EM, irrespective of the type of LNB.

## 1. Introduction

Lyme borreliosis, caused by *Borrelia burgdorferi* sensu lato (s. l.), is the most common tick-borne disease in the northern hemisphere [1,2,3,4,5]. The most frequent initial presentation of Lyme borreliosis is the erythema migrans (EM) skin lesion [6,7,8,9,10]. Extra-cutaneous manifestations of Lyme borreliosis may occur while EM is still present, or they may develop weeks to months after the spontaneous resolution of the skin lesion [6,8,9,10]. An early study in the United States found that 62% of patients with untreated EM later developed Lyme arthritis [11]. In Europe, the consequences of untreated EM are less well defined. However, it appears that additional clinical manifestations are rarer than in the United States and that nervous system involvement is more common than arthritis [10,12].

*Borrelia* enter the skin by tick bite and are thought to disseminate from the skin to other organs. Although hematogenous dissemination is probably the explanation for the development of secondary EM skin lesions and most extracutaneous manifestations [1,9,10], this may not be the case for borrelial meningoradiculoneuritis (Bannwarth’s syndrome), the most common presentation of early Lyme neuroborreliosis (LNB) in adult patients in Europe [13,14,15,16], in which—as was shown in a recent study—retrograde spread of *Borrelia* from skin to the spinal cord via peripheral nerves seems more likely [17]. In that study, the duration of EM prior to diagnosis was significantly longer in patients with borrelial meningoradiculoneuritis than in EM patients without CNS involvement (30 vs. 10 days, *p* < 0.001).

The objectives of the present study were to evaluate if a longer duration of the EM skin lesion is only found in patients with borrelial meningoradiculoneuritis, or if is it also associated with other manifestations of LNB, and to assess potential reasons for the longer duration.

## 2. Materials and Methods

### 2.1. Patients and Definitions

This study is based on clinical information from patients at least 15 years of age evaluated for Lyme borreliosis at the Lyme Borreliosis Outpatient Clinic in Ljubljana, Slovenia, including: (i) 46 patients diagnosed during 2006–2022, who were referred for EM and had a typical EM associated with symptoms/signs of moderate to severe intensity suggesting nervous system involvement, but without radicular pain or cranial neuritis, who consented to a lumbar puncture, and had cerebrospinal (CSF) pleocytosis; (ii) 26 patients referred with cranial neuritis without radicular pain who had EM and CSF pleocytosis; and (iii) 122 patients from the same time period referred for painful meningoradiculoneuritis, who also had EM and CSF pleocytosis. 

These patients represented all patients diagnosed with LNB in the 17-year time period who had an associated EM. The occurrence of EM in our patients with LNB was 56% in patients with borrelial meningoradiculoneuritis and 18% in patients who presented with cranial neuritis (peripheral facial palsy). All of the patients enrolled in the present study had been included in other, more targeted studies on LNB, which also incorporated collection of the basic clinical parameters evaluated in the present study.

These patients were assessed for age, sex, information on having received antibiotics to which borrelia are susceptible in vitro, location of EM on difficult to visualize anatomic locations (back of the head, neck, trunk, thigh, and knee), as well as the duration and largest diameter of the EM skin lesion at the time of diagnosis (since the spread of EM may be influenced by antibiotic treatment, information on diameter was assessed only for those cases for whom antibiotics had not been given up to the point of assessment), and the presence of symptoms at the site of the EM skin lesion. A structured questionnaire was used to assess the clinical data. Since patients with EM and CSF pleocytosis, with or without cranial neuritis but with no radicular pain, did not differ significantly, these 2 groups were combined into a single group entitled borrelial meningitis with or without cranial neuritis (Table 1).

Appropriate antibiotics were defined as those that show in vitro anti-borrelial activity and which had been proven to be effective in clinical studies; these antibiotics included doxycycline, amoxicillin, penicillin, cefuroxime, and azithromycin.

Information on the duration of EM in patients without neurologic involvement was available for 12,189 out of 12,384 patients diagnosed with typical EM at our Lyme Borreliosis Outpatient Clinic during the period 1990–2014. Of these, 9409 had not received any antibiotic treatment up to the time of examination.

Patients diagnosed with EM at our Lyme Borreliosis Outpatient Clinic, who reported moderate to severe symptoms suggestive of nervous system involvement (headache, vertigo, disturbances of sleep, memory or concentration, radicular pain, paresthesias, neck stiffness, and peripheral facial palsy or other cranial nerve involvement [at least one of these symptoms/signs was required to qualify for inclusion in this group]) were offered CSF examination. Those who had CSF pleocytosis (>5 × 10^6^ leukocytes/L) comprised the group of borrelial meningitis, with or without associated cranial neuritis. Patients with radicular pain who fulfilled criteria for borrelial meningoradiculoneuritis were diagnosed to have borrelial meningoradiculoneuritis.

EM was defined as an expanding erythematous skin lesion, with or without central clearing, which developed days to weeks after a tick bite or exposure to ticks in a Lyme borreliosis endemic region and had a diameter of ≥5 cm [18]. Multiple EM was defined as the presence of ≥2 skin lesions, at least 1 of which fulfilled the size criteria (≥5 cm) for a solitary EM. For patients with multiple EM, the location and size of the primary EM were evaluated.

Primary EM was defined as an EM that appeared at the site of the tick bite. If no tick bite was recalled, the primary EM was defined as the lesion with the longest duration, or in the case of the same or uncertain duration, the one with the largest diameter.

The location of EM on difficult to visualize anatomic sites was defined as an EM skin lesion on the back of the head, neck, trunk, thigh, or knee.

The onset of EM was interpreted as the day when the skin lesion was first seen by the patient; the duration of EM in days was based on the number of days from the onset of the skin lesion until the initial visit to the Lyme Borreliosis Outpatient Clinic.

Borrelial meningoradiculoneuritis was defined based on the presence of three criteria: (1) radicular pain, (2) CSF pleocytosis, and (3) demonstration of borrelial (CNS) infection by intrathecal synthesis of borrelial antibodies and/or by isolation of borrelia on culture of CSF and/or by the presence of an EM skin lesion. Only the subgroup of patients with borrelial meningoradiculoneuritis who had EM, however, qualified for the present study.

Borrelial meningitis was diagnosed in (i) patients who were referred for EM and had a typical EM associated with symptoms/signs of moderate to severe intensity suggesting nervous system involvement, but without radicular pain or cranial neuritis, who consented to a lumbar puncture and had cerebrospinal (CSF) pleocytosis; and in (ii) patients referred with cranial neuritis without radicular pain who had EM and CSF pleocytosis. Diagnostic criteria for, and clinical categorization of, Lyme neuroborreliosis in patients enrolled in the present study are shown in Table 2.

### 2.2. Microbiologic Evaluation

Up to 2011, IgM and IgG antibodies to *B. burgdorferi* s. l. in serum and CSF were determined by an indirect immunofluorescence assay. A local isolate of *B. afzelii* was used as an antigen. Reactivity at serum dilutions of 1:256 or higher (or 1:4 or higher in CSF) was interpreted as positive, based on the results of a control group from the same geographic region [19]. Later, an indirect chemiluminescence immunoassay (LIAISON^®^, Diasorin, Saluggia, Italy) with the recombinant antigens OspC and VlsE was used for the detection of IgM antibody, whereas VlsE alone was used for the detection of IgG antibody. The results were interpreted according to the manufacturer’s instructions. 

Intrathecal borrelial antibody synthesis was determined as described by Reiber and Peter [20]: an antibody index >1.4 was considered indicative of intrathecal borrelial antibody production using the indirect chemiluminescence immunoassay and >2 using the indirect immunofluorescence assay.

Cultivation of borrelia from CSF was performed as described previously [21].

### 2.3. Statistical Analysis

Continuous variables were summarized using median values and interquartile ranges (IQRs) and discrete variables using counts and percentages (with 95% confidence intervals, CIs). For discrete variables, all comparisons between groups were based on a Fisher’s exact test. Differences in the duration of EM (in days) and diameter of the EM skin lesion were compared using the Wilcoxon rank-sum test. *p*-values < 0.05 were considered statistically significant.

All statistical analyses were performed using R software (v. 4.3.1) [22].

## 3. Results

The present study included 194 patients with EM and CSF pleocytosis: 122 had radicular pain and fulfilled the criteria for borrelial meningoradiculoneuritis, while 72 were categorized as having aseptic meningitis alone (46 patients) or with cranial neuritis (26 patients, of whom 20 patients had facial palsy and six patients had involvement of other cranial nerve(s)), but without radiculitis. In 131/194 (67.5%) patients, the diagnosis of *Borrelia* infection was established by demonstration of intrathecal synthesis of borrelial antibodies and in 18/194 (9.3%) by a positive CSF *Borrelia* culture; 10 (5.2%) patients had a positive CSF *Borrelia* culture without intrathecal borrelial antibody synthesis, while in 53/194 (27.3%) patients borrelial infection was based solely on the presence of EM.

A comparison of EM patients with borrelial meningoradiculoneuritis and those with CSF pleocytosis without radicular pain (Table 3) revealed that patients with radicular pain were significantly older in age (median 60, IQR 52–67 years vs. median 46, IQR 31–57 years; *p* < 0.001) and had a larger EM skin lesion (median 28, IQR 20–37 cm vs. median 15, IQR 10–20 cm; *p* < 0.001), while the other parameters assessed in the present study, including the duration of EM prior to evaluation for LNB (median 31 vs. 24 days; *p* = 0.470), were not significantly different.

However, the duration of EM in the total group of patients with LNB (median 30, IQR 9–49 days), as well as in each of the two subgroups (median 31, IQR 10–49 in patients with borrelial meningoradiculoneuritis; median 24, IQR 7–53 days in patients without radicular pain), was statistically significantly (*p* < 0.001) longer than in the 12,189 patients with EM without neurologic symptoms for whom this information was available (median 10, IQR 4–24 days). Furthermore, the largest diameter of the EM skin lesion in the total group of untreated patients with LNB (median 20, IQR 15–35 cm) was statistically significantly (*p* < 0.001) greater than in the 9409 patients with EM without neurologic symptoms who had not received antibiotics previously (median 13, IQR 8–20 cm) (Table 4). However, when comparing the EM diameters in each of the two LNB subgroups with the corresponding diameters of EM in patients without neurologic symptoms (median 13, IQR 8–20 cm), a statistically significant difference (*p* < 0.001) was found only for patients with borrelial meningoradiculoneuritis (median 28, IQR 20–37 cm) and not for patients without radicular pain (median 15, IQR 10–20 cm; *p* = 0.291). 

In total, 124/194 (63.9%) patients had not received any antibiotic, while 70/194 (36.1%) patients had received at least one dose of an appropriate antibiotic for treatment of EM (57 azithromycin, 6 amoxicillin, 3 doxycycline, 2 azithromycin followed by amoxicillin, 2 azithromycin followed by doxycycline), and 49/194 (25.3%) had completed antibiotic therapy for EM prior to the diagnosis of LNB. However, 39 of these 49 (79.6%) had had clinical symptoms suggestive of neurologic involvement before beginning antibiotic treatment (Table 5), and none of these 49 patients had received doxycycline in an adequate dose and duration (14 days) for treatment of early LNB. 

Of 26 patients with peripheral facial palsy and EM, only 4 (15.4%) had received corticosteroids prior to referral.

Hypothetically, EM lesions located in difficult to visualize anatomic locations on the back of the head, neck, trunk, thigh, or knee might have been the primary reason for delayed recognition in the LNB groups. However, the findings of the present study do not strongly support this hypothesis, since EM lesions were present at these sites in a minority of cases: in 39/122 (32.0%) of patients with borrelial meningoradiculoneuritis and in 18/72 (25.0%) patients with neurologic symptoms without radicular pain. However, for 17 out of 194 (8.8%) patients, EM was discovered only at the time of the clinical evaluation for LNB; in this subgroup of 17 patients, 9 (52.9%) had their skin lesion(s) in difficult to visualize locations, while in the subgroup of 177 patients who had noticed the lesion before LNB diagnosis 48 (27.1%) had EM in difficult to notice locations (*p* = 0.029).

## 4. Discussion

The prerequisite for appropriate treatment of Lyme borreliosis is timely diagnosis. EM, which is the initial and by far the most common clinical presentation of Lyme borreliosis [8,9,10,23,24,25], is relatively easy to recognize, if it is visualized and if the person visualizing the skin lesion is familiar with the typical appearance and course of an EM skin lesion. We recently found that at diagnosis the duration of EM in patients with borrelial meningoradiculoneuritis was significantly longer than in EM patients without neurologic involvement (median 30 vs. 10 days; *p* < 0.001) [17]. In the present study, we assessed the characteristics of EM in patients with different neurologic manifestations and evaluated if a longer duration of the EM skin lesion was unique to patients with borrelial meningoradiculoneuritis or if it also occurred in patients with LNB but who did not have radicular pain. Our results revealed no statistically significant difference in the duration of the skin lesion between the two LNB groups. However, the duration of EM prior to diagnosis was significantly longer for patients with borrelial meningoradiculoneuritis and also for patients with CSF pleocytosis without radicular pain than for EM patients without LNB. 

Understanding why EM skin lesions were of longer duration in patients with neurologic involvement is of practical importance, since the earlier recognition of EM would allow for relatively simple and effective antibiotic treatment that would most likely prevent progression to LNB. We hypothesized that a longer duration of EM at the time of examination for LNB is primarily a result of its delayed detection and/or a delay in recognition that a skin lesion was a manifestation of Lyme borreliosis but also evaluated several other potential possibilities.

EM lesions located on the back of the head, neck, trunk, thigh, or knee might contribute to difficulties in detection and could be the explanation for going unnoticed. However, the findings of the present study do not support the premise that difficult to visualize anatomic locations is the primary explanation, since EM lesions were present at these sites in a minority of cases (in 32% of patients with borrelial meningoradiculoneuritis and in 25% of patients with neurologic symptoms without radicular pain). As we do not have information on this parameter in the patients with EM but without neurologic symptoms and since a literature search did not reveal any report on the corresponding findings for patients with EM without neurologic involvement, we cannot evaluate whether the proportion of EM in difficult-to-visualize locations in patients with LNB is higher than in those without neurologic impairment. Nevertheless, the frequency of the skin lesion in difficult to visualize locations was statistically significantly higher (*p* = 0.029) in patients who had not been aware of having had EM prior to evaluation for LNB (9/17, 52.9%), compared to those who had noticed the lesion before LNB diagnosis (48/177, 27.1%).

Finding an EM skin lesion is based on visual detection. However, the presence of any associated symptoms at that skin site can alert the patient that something is wrong and encourages more vigilant self-observation. In the present study, 44% of patients with borrelial meningoradiculoneuritis and 48% of patients with LNB not associated with radicular pain reported having had symptoms at the site of the EM. In the latter group, 4/72 (5.6%), i.e., 4/20 with peripheral facial palsy, had received corticosteroids prior to referral which might have impacted symptoms at the site of the EM. Nevertheless, these proportions are comparable to those found in Slovenian patients with EM, but without symptoms/signs of neurologic involvement; approximately one-half of the latter patients had mild itching, burning, and/or pain at the site of EM [26,27,28]. 

Although the longer duration of EM prior to treatment in patients who have nervous system involvement is most probably multifactorial, the findings of the present study clearly show that patients with EM who later develop borrelial meningoradiculoneuritis or other manifestations of LNB are likely to represent a somewhat specific group in terms of EM recognition and in regard to understanding the importance of the early treatment of EM (which predominantly depends upon knowledge). In 9% of our patients, EM was discovered only at the time of diagnosis of LNB (at that time the median largest diameter was 25 cm, and none of them had been treated with antibiotics); 53% of this group of patients had their skin lesion(s) at difficult to visualize locations. The others (91%) were aware of having had a skin lesion for a median duration of 30 days. Despite awareness of the skin lesion, almost two-thirds of these subjects did not receive any antibiotic treatment. Only approximately one-quarter of study subjects had completed the appropriate 10–14-day course of antibiotic treatment for EM before the diagnosis of LNB and could therefore be interpreted to represent treatment failure. However, almost 80% of the patients who had received antibiotic treatment already had symptoms suggestive of the presence of LNB at the time of initiating the antibiotic treatment, and none of them had received doxycycline in an adequate dose for 14 days, as recommended for the treatment of early LNB in Europe [29]. The large majority of untreated patients appreciated the presence of skin redness but did not realize that their skin redness was EM and/or were not aware that it should be treated promptly to prevent the progression of infection or simply ignored the risk, while a few (<5%) were misdiagnosed by their personal physicians.

These data suggest where improvements could be made or are needed. On the part of the physician, directed questions about skin lesions and a thorough skin examination of the whole body would be necessary. Our data show that approximately 9% of referred patients did not receive this basic health care approach, as EM was first discovered during the examination at our Lyme Borreliosis Outpatient Clinic, despite having already been evaluated by their family physician. The general population will need to be persistently encouraged to systematically examine their skin for several weeks in the event of a tick bite or exposure to ticks and to consult a physician regarding further action in the event of skin redness. With a known diagnosis of EM, treatment with antibiotics is reasonable and necessary due to the faster disappearance of EM skin lesions and accompanying symptoms, and above all to prevent the spread of infection and progression of the disease, i.e., the appearance of extracutaneous signs of LB. 

In the present study, the data were obtained prospectively using a structured questionnaire; however, in the majority of patients, information on the duration of EM was obtained retrospectively and was based on subjective assessment of the duration of EM by patients, which might affect the results due to recall bias. One of the limitations of the present study is that information on symptoms at the site of the EM skin lesion (local symptoms) was available for only 92/194 (47%) patients. Furthermore, since only patients at least 15 years of age were included in this study, the results may not be applicable to young children. In addition, while the findings are representative of Slovenia and likely of Central Europe and probably other parts of Europe, they are not applicable to North America, where Lyme borreliosis is nearly exclusively caused by *B. burgdorferi*, rather than the *Borrelia* species usually responsible for LNB in Europe [8,9,10,12,21], and borrelial meningoradiculoneuritis is rare [12]. Finally, since all of the patients were Caucasian, we did not assess the potential impact of a darker skin color on the timely recognition of an EM skin lesion; a darker skin color may be associated with the development of extracutaneous manifestations (and which is presumably due to failure to recognize an EM skin lesion), based on data from studies conducted in the USA [30,31,32].

## 5. Conclusions

The results of the present study showed that the duration of the EM skin lesion in EM patients with LNB is longer than in patients with just EM, irrespective of the type of LNB. Furthermore, the findings of the present study also suggest a lack of awareness that a skin lesion is in fact EM and therefore a manifestation of Lyme borreliosis and that delaying antibiotic treatment for EM may lead to the development of other clinical manifestations, such as LNB. Thus, the main message from this study besides failing to appreciate having an EM is that such delays in the treatment of EM may have consequences.

## Figures and Tables

**Table 1:** Information for patients with erythema migrans (EM) and cerebrospinal fluid pleocytosis (CSF) but without radicular pain: comparison of findings in patients with peripheral facial palsy or other cranial nerve involvement (cranial neuritis) and patients without cranial neuritis (aseptic meningitis).

EM with CSF Pleocytosis, No Radicular Pain			
Variables	Aseptic meningitis n = 46	Cranial neuritis ^a^ n = 26	*p*-value
Age	47 (30–58)	46 (36–56)	0.765
Sex, female	20 (43.5%, 28.9–58.9%)	10 (38.5%, 20.2–59.4%)	0.868
Difficult to visualize anatomic locations ^b^	13 (28.3%, 16.0–43.5%)	5 (19.2%, 6.64–39.4%)	0.571
Duration of EM (days) ^c^	24 (7–47)	25 (7–56)	0.883
Size of EM at examination for patients without previous antibiotic therapy (cm) ^d^	16 (13–20) ^e^	11 (10–23) ^f^	0.168
Local symptoms ^g^	9/19 (47.4%, 24.5–71.1%)	5/10 (50.0%, 18.7–81.3%)	0.600

Data are shown as ratio (%, 95% confidence interval) or median (interquartile range). ^a^ Twenty patients had facial palsy and six patients had involvement of other cranial nerve(s). ^b^ The back side of head, neck, trunk, thigh, or knee. ^c^ Days from the date that EM was noticed until the examination for Lyme neuroborreliosis. ^d^ In patients with multiple EM skin lesions, the largest diameter of the primary EM was used. Primary EM was defined as EM that appeared at the site of the tick bite. If no tick bite was recalled, the primary EM was defined as the lesion with the longest duration, or in the case of the same or uncertain duration, the one with the largest diameter. ^e^ Information available for 18 patients. ^f^ Information available for 12 patients. ^g^ Symptoms at the site of the EM skin lesion (itching, burning, and/or pain).

**Table 2:** Diagnostic criteria for and clinical categorization of Lyme neuroborreliosis in patients enrolled in the present study.

Diagnostic Criteria ^a^	Neurologic Symptoms/Signs Suggestive of Lyme Neuroborreliosis ^b^		
	CSF Pleocytosis		
	Demonstration of Borrelial Infection ^c^		
Clinical categorization	Radicular pain	No radicular pain	
	Borrelial meningoradiculoneuritis (Bannwarth’s syndrome) n = 122	Borrelial meningitis n = 72	
		Cranial neuritis n = 26	No cranial nerve involvement n = 66

^a^ All three criteria are mandatory. ^b^ Neurologic symptoms/signs with a duration of <6 months and with no other obvious explanation. ^c^ Demonstration of borrelial (CNS) infection by intrathecal synthesis of borrelial antibodies and/or by isolation of borrelia on culture of CSF and/or by the presence of an erythema migrans skin lesion. All patients (194/194) included in the present study had erythema migrans, 131/194 (67.5%) had intrathecal borrelial antibody synthesis, and 18/194 (9.3%) had a positive CSF *Borrelia* culture; 10 (5.2%) patients had a positive CSF *Borrelia* culture without intrathecal borrelial antibody synthesis, while in 53/194 (27.3%) patients documentation of borrelial infection was based solely on the presence of an erythema migrans skin lesion.

**Table 3:** Basic information on patients with erythema migrans (EM) and cerebrospinal fluid (CSF) pleocytosis: comparison of findings for those with borrelial meningoradiculoneuritis and for those without radicular pain.

EM with CSF Pleocytosis			
Variables	No radicular pain, n = 72 (Borrelial meningitis +/− cranial neuritis)	Radicular pain, n = 122 (Bannwarth´s syndrome)	*p*-value
Age	46 (31–57)	60 (52–67)	**<0.001**
Sex, female	30 (41.7%, 30.2–53.9%)	59 (48.4%, 39.2–57.6%)	0.450
Difficult to visualize anatomic locations ^a^	18 (25.0%, 15.6–36.6%)	39 (32.0%, 23.8–41.0%)	0.386
Duration of EM (days) ^b^	24 (7–53)	31 (10–49)	0.470
Size of EM at examination for patients without previous antibiotic therapy (cm) ^c^	15 (10–20) ^d^	28 (20–37) ^e^	**<0.001**
Local symptoms ^f^	14/29 (48.3%, 29.4–67.5%)	28/63 (44.4%, 31.9–57.5%) ^g^	0.906

Data are shown as a ratio (%, 95% confidence interval) or median (interquartile range). Bold is used for *p*-values < 0.05. ^a^ The back side of neck, trunk, thigh, or knee. ^b^ Days from the date that EM was noticed until the examination for Lyme neuroborreliosis. ^c^ In patients with multiple EM, the largest diameter of primary EM was employed. Primary EM was defined as the EM that appeared at the site of the tick bite. If no tick bite was recalled, the primary EM was defined as the lesion with the longest duration, or in the case of the same or uncertain duration, the one with the largest diameter. ^d^ Information available for 30 patients. ^e^ Information available for 73 patients. ^f^ Symptoms at the site of EM skin lesion (itching, burning, and/or pain). ^g^ For patients with borrelial meningoradiculoneuritis prior to the onset of radicular pain.

**Table 4:** Findings in patients with erythema migrans and Lyme neuroborreliosis in comparison to findings in patients with erythema migrans without neurologic symptoms.

Variables	EM with LNB, n = 194	EM without Neurologic Symptoms, n = 12,384	*p*-Value
Age	56 (45–66)	49 (37–59)	**<0.001**
Sex, female	89 (45.9%, 38.7–53.2%)	7947 (64.2%, 63.3–65.0%)	**<0.001**
Difficult to visualize anatomic locations ^a^	57 (29.4%, 23.1–36.3%)	Not available	
Duration of EM (days) ^b^	30 (9–49)	10 (4–24) ^c^	**<0.001**
Size of EM at examination for patients without previous antibiotic therapy (cm) ^d^	20 (15–35) ^e^	13 (8–20) ^f^	**<0.001**
Local symptoms ^g^	42/92 (45.7%, 35.2–56.4%)	6491/12,351 (52.6%, 51.7–53.4%) ^h^	**<0.001**

Data are shown as a ratio (%, 95% confidence interval) or median (interquartile range). Boldface is used for *p*-values < 0.05. LNB, Lyme neuroborreliosis; EM, erythema migrans. ^a^ The back side of neck, trunk, thigh, or knee. ^b^ Days from the date that EM was noticed until the examination for Lyme neuroborreliosis. ^c^ Information available for 12,189 patients. ^d^ In patients with multiple EM, the largest diameter of the primary EM was used. Primary EM was defined as the EM that appeared at the site of the tick bite. If no tick bite was recalled, the primary EM was defined as the lesion with the longest duration, or in the case of the same or uncertain duration, the one with the largest diameter. ^e^ Information available for 103 patients. ^f^ Information available for 9409 patients. ^g^ Symptoms at the site of EM skin lesion (itching, burning, and/or pain). ^h^ For patients with borrelial meningoradiculoneuritis prior to the onset of radicular pain.

**Table 5:** Characteristics of patients with erythema migrans (EM), for whom the skin lesion was observed before or at the time of diagnosis of Lyme neuroborreliosis (LNB): comparison of findings in patients with borrelial meningoradiculoneuritis versus those with LNB but without radicular pain.

EM with CSF Pleocytosis			
Variables	No radicular pain, n = 72 (Borrelial meningitis +/− cranial neuritis)	Radicular pain, n = 122 (Bannwarth´s syndrome)	*p*-value
**Observation of EM before diagnosis of LNB (n = 177)**			
Number of patients (%)	67 (93.1%, 84.5–97.7%)	110 (90.2%, 83.5–94.8%)	0.671
Duration of EM (days) since first observed	26 (9–55)	34 (17–50)	0.227
Size ^a^ of EM on examination in patients without previous antibiotic therapy (cm)	15 (11–22) ^b^	25 (20–36) ^c^	**0.001**
Difficult to see location of EM ^d^	16 (23.9%, 14.3–35.9%)	32 (29.1%, 20.8–38.5%)	0.561
Previous antibiotic treatment ^e^	31 (46.3%, 34.0–58.9%)	39 (35.5%, 26.6–45.2%)	0.205
Completed adequate antibiotic therapy for EM ^f^	19 (28.4%, 18.0–40.7%)	30 (27.3%, 19.2–36.6%)	0.987
**Observation of EM only at time of diagnosis of LNB (n = 17)**			
Number of patients (%)	5 (6.9%, 2.3–15.5%)	12 (9.8%, 5.2–16.6%)	0.671
Diameter^a^ of EM on examination (cm) ^g^	9 (7–15)	36 (21–50)	**0.009**
Difficult to see location of EM ^d^	2 (40.0%, 5.3–85.3%)	7 (58.3%, 27.7–84.8%)	0.437
Previous antibiotic treatment ^e^	0 (0%, 0–52.2%)	0 (0%, 0–26.5%)	NA

Data are the number (%, 95% confidence interval) or median value (interquartile range). Bold is used for *p*-values < 0.05. Abbreviations: EM, erythema migrans; CSF, cerebrospinal fluid; LNB, Lyme neuroborreliosis; NA, not applicable. ^a^ Largest diameter of erythema migrans skin lesion. ^b^ Information available for 26 patients. ^c^ Information available for 61 patients. ^d^ The back side of head, neck, trunk, thigh, or knee. ^e^ Antibiotics to which borrelia are susceptible in vitro. ^f^ Adequate antibiotic therapy in regard to choice of drug, dosage, and duration. ^g^ None of these patients had received previous antibiotic treatment.

## Data Availability

The data presented in this study are available within the article.
